# Analysis of RNA Expression Specificity and Commonality in Commonly Used Tool Cells and Multiple Tissues of Pigs

**DOI:** 10.3390/biom16030448

**Published:** 2026-03-17

**Authors:** Huan Yang, Chunlei Zhang, Xiaohuan Chao, Jiahao Chen, Yuan Ding, Bo Zhou

**Affiliations:** College of Animal Science and Technology, Nanjing Agricultural University, Nanjing 210014, China; 2024205016@stu.njau.edu.cn (H.Y.); 2020105039@stu.njau.edu.cn (C.Z.); 2021205020@stu.njau.edu.cn (X.C.); 2023205025@stu.njau.edu.cn (J.C.); 2025205018@stu.njau.edu.cn (Y.D.)

**Keywords:** pig, cell lines, tissues, RNA, ubiquitously expressed genes

## Abstract

An increasing number of studies have focused on the redundant roles of genes in various cellular processes. For instance, 37,000 and 127,300 publications are associated with P53 and Tumor Protein 53 (TP53) respectively, and numerous other genes are also repeatedly interpreted like them. Thus, it is crucial to reduce such non-essential duplicated studies. In this study, RNA sequencing (RNA-seq) data of 6 commonly used tool cell lines and 43 tissue types from pigs were analyzed. The results indicated that genes relatively highly or specifically expressed in each cell type most likely perform that cell’s primary function. Specifically, such genes in skeletal muscle cells mainly regulate skeletal muscle structure, differentiation and development, with similar phenomena seen in the other 5 cell types. In addition, RNA expression levels of genes show high similarity and commonality between cells and tissues, with a total of 4117 ubiquitously expressed genes screened out overall. Meanwhile, embryonic samples display the largest number of RNA-specific expressed genes and the strongest tissue specificity. In conclusion, investigating highly and specifically expressed genes across cells, tissues and organs enables more efficient identification of core functional genes, whereas cataloging ubiquitously expressed genes in a species helps reduce redundant and unnecessary gene functional characterization.

## 1. Introduction

Due to their advantages of maximally simulating the real in vivo state and high feasibility, cells are widely used as models for in vitro experimental validation. In porcine research, widely studied traits include reproductive performance, growth rate, fat deposition, lean meat percentage, feed conversion rate, and so on. Molecular verification of these traits often employs tool cells such as skeletal muscle cells (SKMC), ovarian granulosa cells (OGC), Porcine Kidney-15 (PK15) cells, intestinal porcine epithelial cell line-J2 (IPEC-J2), and porcine adipocytes (PAC). Firstly, lean meat percentage is a key economic trait in pig production. During pig growth, skeletal muscle cells increase mass through proliferation and hypertrophy, and the genes governing these functions are regulated at multiple molecular levels. For example, DNA methylation [1], single nucleotide polymorphisms (SNPs), transcription factor (TF) binding, Post transcriptional mechanisms, including mRNA processing, transport, and stability, as well as microRNA mediated regulation, affect gene expression involved in muscle development [2]. Similarly, other traits such as fat deposition are regulated by multiple genes. For instance, the Adiponectin (*ADIPOQ*) gene, which reduces fat accumulation, is implicated in Alzheimer’s disease, breast cancer, coronary artery disease, and bone marrow development [3,4,5,6]. *ADIPOQ* also exhibits associations with breast cancer through SNPs, while its protein level regulation contributes to bone marrow and coronary artery development through distinct mechanisms [7]. In neurological research, cells expressing specific neural genes such as astrocytes, microglia, and oligodendrocytes are commonly used to investigate gene interactions. Neural stem cell and astrocyte transplantation has shown potential in improving cognitive function in patients with multiple sclerosis, although early trials have reported minor cerebrospinal fluid differences [8,9]. With the advent of single cell sequencing, the identified cell populations in the central nervous system have expanded, adding further complexity to the study of gene and cell relationships [10]. In the process of studying corresponding traits using various cells, a large number of ubiquitously expressed genes have developed numerous functions. Cell or tissue specific genes are overshadowed by these genes, which has caused certain obstacles to the classification of gene functions.

In the literature on genes of various animals, some researchers tend to select relatively popular genes among differentially expressed genes for study, which directly leads to the phenomenon that the same gene or multiple genes are subject to repeated and excessive interpretation in different cells becoming increasingly common. For example, overexpression of the cluster of differentiation 164 (*CD164*) gene has been shown to promote proliferation in normal human astrocytes, bladder cancer cells, ovarian surface epithelial cells, hematopoietic stem cells, lung tumor cells, and colon cancer cells [11,12,13]. This raises the question of whether *CD164* overexpression or inhibition exerts similar effects on other human cell types. As of December 2025, according to search results from NCBI alone, there are a total of 3765 publications concerning the growth factor receptor-bound protein 2 (*GRB2*) gene, while 36,031 publications are related to the *TP53* (tumor protein 53) gene. When including publications indexed in other databases, the total number of various types of literature associated with these genes is far greater than these figures. Extensive unnecessary repetitive interpretations have resulted in significant data redundancy. There are many more genes that have been repeatedly studied, just like *TP53*. Therefore, if a gene participates in regulating multiple cellular traits, can we consider it a ubiquitously expressed fundamental gene?

Nearly 150 microRNAs (miRNAs) have been involved in regulating the proliferation of ovarian granulosa cells. However, which one is the core? For miRNAs, which are relatively small in number, the phenomenon of multiple miRNAs regulating cell proliferation has already emerged, so what about mRNAs, which are relatively larger in number? And what about non-coding RNAs, such as lincRNAs, circRNA, siRNA, piRNA, snoRNA, snRNA, and eRNA [14]? These are only some sequencing approaches at the RNA level; there are also other sequencing methods targeting the genome, epigenome, and metagenome, as well as metabolomic. However, most sequencing approaches ultimately boil down to investigating how changes in gene expression affect phenotypic traits. When sequencing data from different levels are integrated, they further expand the functional scope of genes, for example, through combinations of metabolomics and transcriptomics, among many other multiomics combinations [15]. When considering the RNA level combined with the DNA and protein levels, the regulatory network of the entire cellular system becomes far more complex and cumbersome. The excessive repeated validation of the same gene across different cells has made genetic research tedious and inefficient.

This study analyzes the specificity and commonality of RNA expression based on RNA sequencing data from six commonly used tool cells and 43 different tissues of pigs, aiming to clarify the genetic commonalities of the pig genome and lay a solid foundation for reducing redundancy and unnecessary experimental validation.

## 2. Materials and Methods

### 2.1. Sources and Download of Sequencing Data

In this study, RNA sequencing (RNA-seq) data of porcine cells and tissues were retrieved from the NCBI Sequence Read Archive (SRA; https://www.ncbi.nlm.nih.gov/sra, accessed on 22 April 2025). Double-stranded RNA-seq datasets generated using the Illumina high-throughput sequencing platform and with comparable sequencing depth were selected. The accession numbers (PRJNA) obtained from the SRA search results were entered into the European Nucleotide Archive (ENA; https://www.ebi.ac.uk/ena/browser/home, accessed on 26 April 2025) to verify sequencing depth and data quality. Datasets meeting the screening criteria were downloaded to the local server via the Ascp (v3.9.1) package in Aspera (v3.9.1). The downloaded porcine cellular RNA-seq datasets included porcine adipocytes (PAC, *n* = 9; PRJNA883198), skeletal muscle cells (SKMC, *n* = 6; PRJNA594300), PK15 cells (PK15, *n* = 8; PRJNA773967), and intestinal porcine enterocyte cell line (IPEC-J2, *n* = 9; PRJNA1180182). RNA-seq data of The downloaded porcine tissue RNA-seq datasets included adipose tissue (PRJEB23654, *n* = 3), skeletal muscle tissues (PRJNA736938, *n* = 2), bone marrow, rib, and lymph node (PRJNA1104148, *n* = 1), stomach, small intestine, large intestine, and testis (PRJNA980289, *n* = 1), heart, liver, spleen, lung, and kidney (PRJNA1215451, *n* = 2), ovary (PRJNA562823, *n* = 2), blood (PRJNA1224715, *n* = 2), embryos (PRJNA854394, *n* = 2), and deep embryo (PRJNA882399; *n* = 1), aorta, tonsil, trachea, urinary bladder, cartilage-joint, colon, esophagus, joint-adjacent, oral mucosa, peritoneum, pleura, rectum, tongue-surface, veins and penis (CNP0001361, *n* = 1), black skin and white skin (PRJNA903765, *n* = 1), gallbladder (PRJNA953778, *n* = 1), duodenum (PRJNA271310, *n* = 1). RNA-seq data of brain tissues (frontal lobe, hypothalamus, hypophysis, parietal lobe and temporal lobe) were obtained from our laboratory.

### 2.2. Quality Control, Filtration, Alignment, and Quantification of Sequencing Data

Quality assessment of all RNA-seq datasets was performed using FastQC (v0.12.1) on the Java platform, with a required quality control rate exceeding 95%. Parameters such as the base quality score (Q30), average read quality distribution, and sequence composition were evaluated. Samples that did not meet the quality requirements were excluded from subsequent analyses. Sequence trimming and filtering were conducted in a Python 3.12 environment using Cutadapt (v4.9; https://cutadapt.readthedocs.io/en/stable/, accessed on 26 April 2025) developed by Marcel Martin. Adapter sequences and low-quality reads were removed, and only high-quality sequences were retained for downstream processing. The porcine reference genome file Sus_scrofa.Sscrofa11.1.dna.toplevel.fa.gz was downloaded from Ensembl (https://ftp.ensembl.org/pub/release-113, accessed on 6 May 2025). Genome indexing was performed using HISAT2 (v2.2.1), followed by alignment of the cleaned reads to the reference genome. The resulting temporary BAM files were merged and processed using SAMtools (v1.21). The corresponding genome annotation file Sus_scrofa.Sscrofa11.1.113.gtf.gz was also obtained from Ensembl. Gene-level quantification was carried out using Subread (v2.0.8) to generate the final gene expression count matrix for subsequent analyses.

### 2.3. Weighted Gene Co-Expression Network Analysis

All analyses of cell related data were conducted in RStudio (v4.4.2). FactoMineR (v2.11), ggplot2 (v3.5.2), ggrepel (v0.9.6) and plyr (v1.8.9) in R were used for principal Component Analysis (PCA) and visualization of the overall distribution of samples. The RNA-seq read counts of the 6 porcine cell types were converted to Fragments Per Kilobase of transcript per Million mapped reads (FPKM) values. The apply function was then used to calculate the Median Absolute Deviation (MAD) for each gene, with the filtering threshold set to 0 (derived from the sequence generated by the seq function) to retain a greater number of genes for downstream analysis. Weighted Gene Co-expression Network Analysis (WGCNA) was performed using the R packages stringi(v1.8.4), WGCNA (v1.73), flashClust (v1.1.2), and iterators (v1.0.14). FPKM expression matrices were imported and filtered, and sample grouping information was integrated. Sample clustering and phenotype visualization were conducted, and outlier samples were removed based on hierarchical clustering results. A power function was applied to transform the gene correlation matrix into an adjacency matrix. When the scale free topology fit index reached 0.85, a soft threshold (β) of 12 was selected for subsequent analyses. A Topological Overlap Matrix (TOM) was then constructed using the following formula:

*ω_ij_* = (*l_ij_* + *a_ij_*)/(min{*k*_i_, *k_j_*} + 1 − *a_ij_*)

where *ω_ij_* denotes the topological overlap measure between gene *i* and gene *j*, *a_ij_* represents the corresponding element in the adjacency matrix (i.e., the power of the correlation coefficient), and *k_i_* and *k_j_* denote the connectivity of genes *i* and *j*, respectively.

The Pearson correlation coefficient between genes was calculated as:

*s_ij_* = cor|(*x_i_*, *x_j_*)|

where *s_ij_* ranges from 0 to 1. The adjacency matrix was then derived using the equation:

*a_ij_* = (*s_ij_*)*β*

The deepSplit parameter, which controls the sensitivity of module detection, was set to 2 by default. Modules were identified using the dynamic tree cutting method with a minimum module size (minModuleSize) of 30. The mergeCutHeight value, representing the threshold for module merging, was set to 0.25.

Finally, Gene Significance (GS) and Module Membership (MM) were calculated. GS represents the correlation between each gene within a module and the associated phenotype, whereas MM quantifies the correlation between an individual gene and its respective module eigengene.

### 2.4. Downstream Analysis of Gene Sets of Modules Significantly Correlated with Various Cell Types

Gene modules that exhibited significant correlations with specific cell types were selected for downstream analysis. The pheatmap (v1.0.13) package in R was used to visualize the expression profiles of characteristic genes through heatmaps, while bar plots were generated using GraphPad Prism (v8.0). The R package org.Ss.eg.db (v3.20.0) (http://www.bioconductor.org/packages/release/data/annotation/html/org.Ss.eg.db.html, accessed on 26 May 2025) was downloaded from the Bioconductor annotation database to obtain the Entrez gene identifiers for *Susscrofa*. Data transformation and organization were performed using the dplyr package of R. Functional enrichment analyses, including Gene Ontology (GO) and Kyoto Encyclopedia of Genes and Genomes (KEGG) pathway analyses, were conducted using the clusterProfiler (v4.14.6) package. Thresholds for significant enrichment were set as pvalueCutoff = 0.05 and qvalueCutoff = 0.05. Gene interaction networks for key GO and KEGG enriched terms were constructed and visualized using Cytoscape (v3.7.2) (https://cytoscape.org/, accessed on 4 June 2025). The enrichment results were further visualized using the ggplot2 package in R. Additionally, the apply function in base R was employed to calculate the mean FPKM expression level of each gene across different cell types.

### 2.5. Cultivation and Morphological Observation of Various Porcine Cells

Porcine ovarian granulosa cells (OGC) were aspirated from ovarian follicles with diameters of 3–5 mm using a sterile syringe. The cells were cultured in nutrient F-12 medium (DMEM/F-12, Gibco, Grand Island, NY, USA) supplemented with 10% fetal bovine serum (FBS; Gibco, Grand Island, NY, USA) at 37 °C in a humidified atmosphere containing 5% CO_2_. 24 h after seeding, cells were gently washed with phosphate-buffered saline (PBS; Solarbio, Beijing, China) and supplied with fresh complete medium. Morphological observations were recorded when the primary OGC reached confluence. PK15 cells were cultured in DMEM medium (Gibco, Crand Island, NY, USA) supplemented with 10% FBS and 1% penicillin-streptomycin (Gibco, Grand Island, NY, USA) under identical incubation conditions. IPEC-J2 cells were maintained using the same culture protocol as PK15 cells. Porcine adipocytes (PAC) were isolated from the dorsal subcutaneous adipose tissue of 7-day-old piglets and seeded into T25 (T25; NEST, Wuxi, China) culture flasks containing complete medium composed of F-12 medium supplemented with 10% FBS and 1% penicillin streptomycin (Gibco, Carlsbad, CA, USA). The cells were washed with PBS and refreshed with new medium every 48 h. When PAC reached approximately 80% confluence, representative fields were selected and photographed. The isolation and culture procedures followed the method described by Zhang et al. [15]. Porcine neuroglial cells (PNGC) were isolated from the brain tissue of 30-day-old pigs and seeded into T25 culture flasks using complete medium consisting of F-12 medium supplemented with 10% FBS and 1% penicillin-streptomycin. Photographs were taken when PNGC cultures reached 80% confluence. The isolation and culture procedures were based on the protocol of Yang et al. [16]. All cultured cell types were photographed under a light microscope at identical magnifications upon reaching confluence to document their morphological characteristics. The animals used in the experiment were all approved by the Animal Experimentation Ethics Committee of Nanjing Agricultural University (Approval No. NJAULLSC2021030).

### 2.6. Analysis of RNA Expression Profiles in Cells, Cellular Components, and Tissues

TheedgeR (v4.4.2), reshape (v0.8.10), and ggplot2 (v3.5.2) packages in R were used to calculate and visualize gene expression distributions. Whole-genome expression heatmaps were generated using the pheatmap package. Raw RNA Count values were converted to Transcripts Per Million (TPM) values using R software, and genes with TPM values greater than 1 across all samples were extracted using R. Functional enrichment analyses and network visualizations of ubiquitously expressed genes were performed using the clusterProfiler (v4.14.6), org.Ss.eg.db (v3.20.0), enrichplot (v1.26.6), aPEAR (v1.0), ggplot2 (v3.5.2), and AnnotationDbi (v.1.68.0) packages in R.

### 2.7. Statistical Analysis

The statistical significance of RNA expression differences among cell types was evaluated using unpaired *t*-tests. For each cell type, more than three biological replicates were included in the sequencing analysis. Data are presented as the mean ± standard error of the mean (SEM), and differences were considered statistically significant at *p* < 0.05.

## 3. Results

### 3.1. Weighted Gene Go-Expression Network Analysis (WGCNA) of 6 Common Porcine Tool Cells

**Figure 1:**
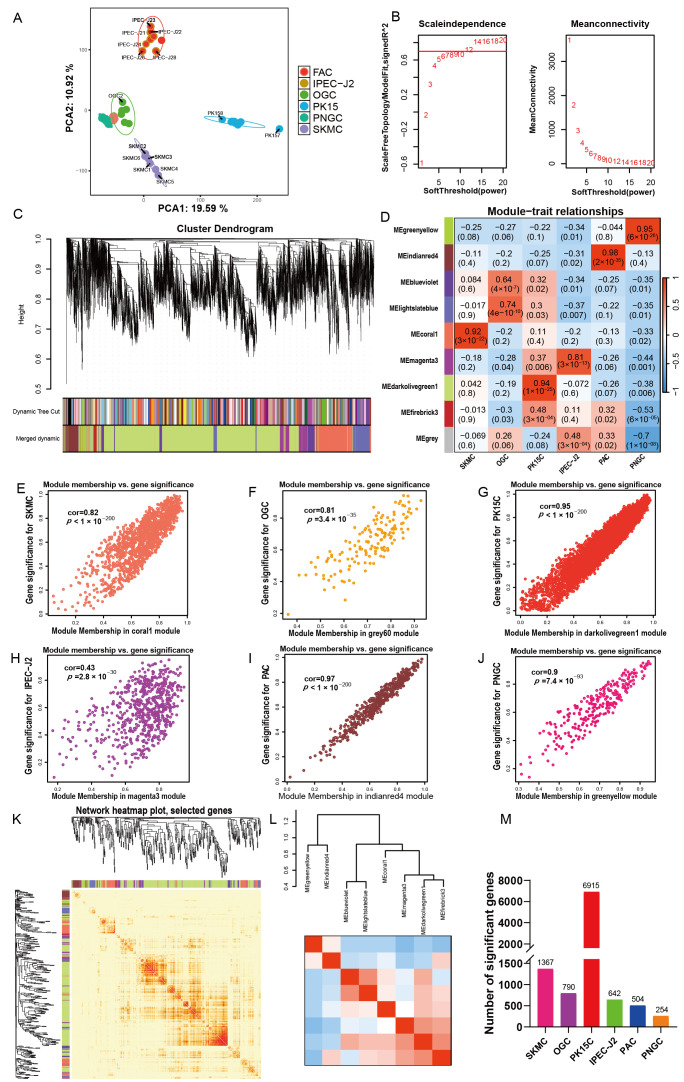
Weighted gene co-expression network analysis of 6 porcine cell types. (**A**) Sample dendrogram and trait heatmap showing clustering of six porcine cell types. (**B**) Determination of the optimal soft-threshold power (β). When the scale free topology fit index reached 0.85, the β was 12. The horizontal axis represents the β, and the vertical axis indicates average connectivity. (**C**) Hierarchical clustering dendrogram constructed from pairwise gene correlations. The lower panel shows module assignment based on weighted correlation coefficients, with genes exhibiting similar expression patterns grouped into the same module. Distinct modules are represented by different colors; gray indicates unassigned genes. (**D**) Heatmap showing correlations between 12 gene modules and six cell types. Numbers outside parentheses represent Pearson correlation coefficients, and those within parentheses represent *p*-values. Red indicates positive correlation, and blue indicates negative correlation. (**E**–**J**) Scatter plots depicting relationships between module eigengenes and cellular phenotypic traits. cor denotes the Pearson correlation coefficient, and statistical significance is indicated by *p* < 0.05. (**K**) Heatmap visualization of the topological overlap matrix (TOM) among all genes. Lighter colors represent lower overlap, whereas darker red indicates higher overlap. Darker blocks along the diagonal correspond to gene modules. The gene dendrogram and module assignments are displayed along the left and top margins. (**L**) Hierarchical clustering dendrogram and heatmap of module eigengenes. The upper part of the figure shows the hierarchical clustering results based on the similarity of module eigengenes, the vertical axis represents the similarity between modules, and the heatmap presents the correlation between modules. (**M**) Bar chart showing the number of module genes significantly associated with each cell type.

Significant differences in reproduction, tolerance, morphology, and size exist among commonly used porcine cell types. To identify gene groups underlying these functional distinctions, weighted gene co-expression network analysis (WGCNA) was performed on six cell types. Sample clustering analysis demonstrated that porcine adipocytes (PAC) and porcine neuroglial cells (PNGC) showed robust within group clustering (Figure 1A). When the scale free network fit index reached 0.85, the optimal soft threshold power (β) was determined to be 12 (Figure 1B). In the merged dynamic of the cluster dendrogram, the module colored Medarkolivegreen1 accounts for the largest proportion (Figure 1C). Six distinct gene modules displayed highly cell specific expression patterns (Figure 1D), with significant correlations between module eigengenes and sample types (*p* < 0.05). Among these, the Pearson correlation coefficients between module eigengenes and genes were 0.81 for skeletal muscle cells (SKMC) and 0.82 for ovarian granulosa cells (OGC) (Figure 1E,F). The IPEC-J2 associated module exhibited a lower correlation coefficient of 0.43 (Figure 1H), whereas those associated with PK15, PAC, and PNGC each showed coefficients ≥ 0.9 (Figure 1G,I,J). The gene clustering heatmap based on the topological overlap matrix indicated numerous modules with high intra-module connectivity (Figure 1K). Hierarchical clustering of module eigengenes revealed four major clusters, with MEblueviolet and MElightslateblue showing the highest similarity, followed by MEdarkolivegreena and MEfirebrick3 (Figure 1L). The distribution of correlated module genes across cell types demonstrated that 6915 genes were associated with PK15, 1367 with SKMC, and 254 with PNGC (Figure 1M and Table S1).

### 3.2. Genes Exhibiting Positive Correlations with Each Cell Type Are Potentially Key to Maintaining the Core Functions of the Corresponding Cell

**Figure 2:**
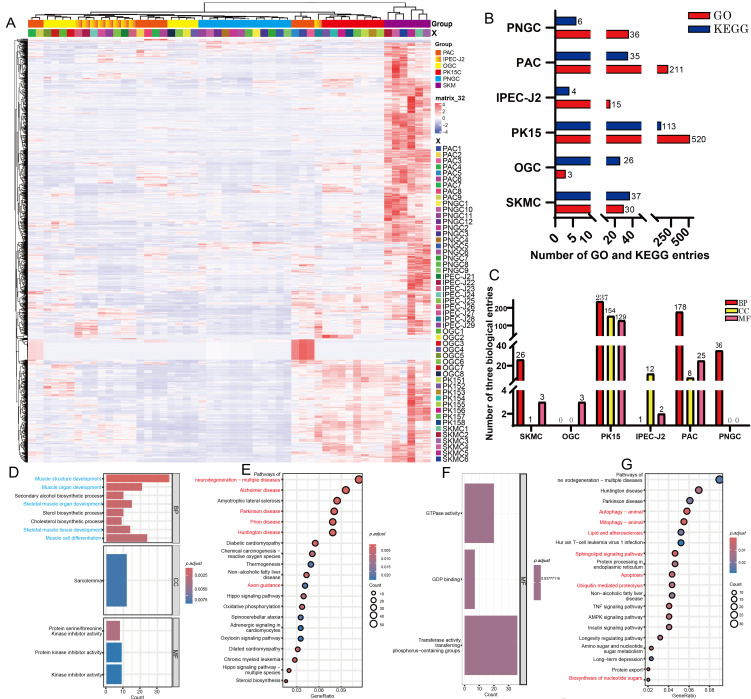
Functional enrichment analysis of genes significantly associated with various porcine cell types. (**A**) Heatmap showing the expression profiles of characteristic genes significantly associated with skeletal muscle cells (SKMC). Red indicates higher gene expression levels, and blue indicates lower expression levels. (**B**) Numbers of Gene Ontology (GO) (Biological Process [BP], Cellular Component [CC], and Molecular Function [MF]) and Kyoto Encyclopedia of Genes and Genomes (KEGG) terms enriched by genes significantly associated with each cell type. (**C**) Counts of enriched BP, CC, and MF GO terms corresponding to gene sets from significant modules across cell types. (**D**,**E**) GO (**D**) and KEGG (**E**) enrichment terms for the gene significantly associated with SKMC. (**F**,**G**) GO (**F**) and KEGG (**G**) enrichment terms for the gene significantly associated with ovarian granulosa cells (OGC), pathways with the same color represent functionally similar pathways.

**Figure 3:**
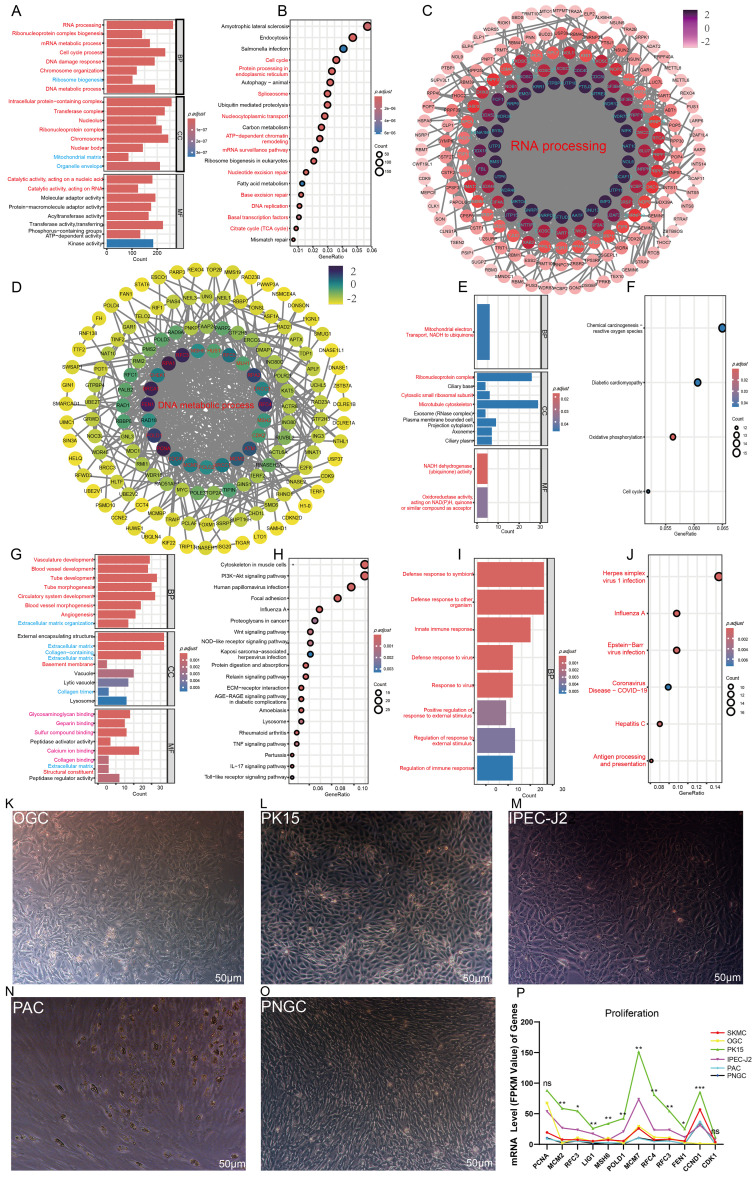
The proliferation capacity of cells and the expression abundance of genomic RNA exhibit a positive correlation. (**A**,**B**) GO (**A**) and KEGG (**B**) enrichment terms for the gene significantly associated with PK15 cells. (**C**) Interaction network of genes significantly associated with PK15 cells and enriched in the DNA metabolic process pathway. The darker the node color, the higher the degree of gene interaction. (**D**) Interaction network of genes significantly associated with PK15 cells and enriched in the RNA processing pathway. The darker the node color, the higher the degree of gene interaction. (**E**,**F**) GO (**E**) and KEGG (**F**) enrichment terms for the gene significantly associated with intestinal porcine enterocyte cell line (IPEC-J2), (**G**,**H**) GO (**G**) and KEGG (**H**) enrichment analyses of the gene set from the MEindianred4 module significantly associated with porcine adipocytes (PAC). (**I**,**J**) GO (**I**) and KEGG (**J**) enrichment analyses of the gene set from the MEgreenyewllow module significantly associated with porcine neuroglial cells (PNGC), pathways with the same color represent functionally similar pathways. (**K**–**O**) Representative microscopic images of porcine ovarian granulosa cells (OGC) (**K**), PK15 cells (**L**), IPEC-J2 (**M**), PAC (**N**), PNGC (**O**) at confluence. Scale bars, 50 μm. (**P**) FPKM values representing the mRNA expression levels of cell proliferation marker genes. Differential analysis was performed on two cell lines: PK15 and IPEC-J2. Asterisks denote significance levels: * *p* < 0.05; ** *p* < 0.01; *** *p* < 0.001. “ns” indicates no significant difference.

To validate the expression patterns of the significant gene sets identified by WGCNA, a cluster heatmap was generated using genes significantly associated with SKMC. The results showed that SKMC replicates clustered closely, with expression patterns distinct from those of the other five cell types, confirming the reliability of the weighted analysis (Figure 2A). Gene Ontology (GO) and Kyoto Encyclopedia of Genes and Genomes (KEGG) enrichment analyses were then performed for gene sets significantly associated with each cell type. GO terms were classified into three categories: Biological Process (BP), Cellular Component (CC), and Molecular Function (MF) (Figure 2B,C). Genes significantly associated with SKMC were primarily enriched in GO terms related to muscle cell differentiation, muscle structure and organ development, and skeletal muscle tissue organization (Figure 2D). KEGG pathway analysis revealed enrichment in neurodegenerative disease pathways, including Alzheimer’s disease, Parkinson’s disease, prion diseases, and Huntington’s disease, as well as axon guidance (Figure 2E). For ovarian granulosa cells (OGC), GO enrichment indicated three MF terms related to energy metabolism (Figure 2F). Corresponding KEGG analysis showed enrichment in apoptosis, autophagy, and certain neurological disease pathways (Figure 2G). The gene set associated with PK15 contains the most abundant terms, including 237 CC terms, 154 MF terms, 129 BP terms, and 139 KEGG pathways (Figure 2B,C). These genes were enriched in processes related to cell mitosis and proliferation, including DNA metabolism and repair, RNA and mRNA processing, protein activity, chromatin organization, and cell cycle regulation (Figure 3A,C,D). KEGG results further confirmed enrichment in pathways associated with cellular proliferation and mitotic activity (Figure 3B). Genes associated with intestinal porcine enterocyte cell line (IPEC-J2) were mainly enriched in three nicotinamide adenine dinucleotide (NADH) dehydrogenase related pathways (Figure 3E), while KEGG analysis indicated involvement in reactive oxygen species and oxidative phosphorylation pathways (Figure 3F). Genes associated with porcine adipocytes (PAC) are mainly enriched in pathways related to tubular morphogenesis at the Biological Process (BP) level, while at the Cellular Component (CC) level, these genes are mainly enriched in pathways related to extracellular matrix membrane structures (Figure 3G,H). For porcine neuroglial cells (PNGC), GO analysis revealed enrichment in immune related pathways, and KEGG results indicated associations with disease related signaling (Figure 3I,J), reflecting the dual protective and immune roles of glial cells. The observation that specifically or highly expressed genes in each cell type largely correspond to those governing the cell’s primary function is unlikely to be coincidental.

### 3.3. The Total RNA Expression Level of Cells Is Positively Correlated with Their Proliferative Capacity and Stability

Based on the preceding analyses, genes significantly associated with PK15 were enriched in pathways related to cell mitosis and proliferation. To further verify the relationship between cell proliferation rate and gene expression abundance, five types of porcine cells were cultured, and their external morphological characteristics were observed under a microscope. OGC displayed distinct cellular boundaries, irregular shapes, and considerable morphological diversity (Figure 3K). PK15 cells exhibited clear boundaries, intact and smooth membranes, and polygonal or short fusiform shapes. They grew adherently and were densely arranged, forming compact monolayers (Figure 3L). Intestinal porcine enterocyte cell line (IPEC-J2) has typical epithelial morphological characteristics, exhibit a polygonal structure, with clear and tight intercellular junctions, and often form a monolayer cobblestone shaped structure, similar to PK15 cells (Figure 3M). Porcine adipocytes (PAC) and neuroglial cells (PNGC) both appeared elongated and fusiform, with PNGC displaying particularly small cell bodies (Figure 3N,O). Overall, the cell lines PK15 and IPEC-J2 were morphologically fuller and more uniform than primary cells. Among the six cell types, the total mRNA read counts of genes in PK15 cells were significantly higher than those in the second-highest SKMC and IPEC-J2 cell lines (Supplementary Figure S1). Further analysis of marker genes associated with cell proliferation showed that except for the FPKM values of *CDK1* and *PCNA*, which show no significant difference between PK15 and IPEC-J2, the FPKM values of all other proliferation-related genes in PK15 are significantly higher than those in IPEC-J2; compared with the other five cell types, the mRNA levels of proliferation-related marker genes in PK15 are the highest among the six cell types (Figure 3P).

### 3.4. The RNA Expression in the Commonly Used Tool Cells and Tissues of Pigs Has Highly Identical Commonalities

**Figure 4:**
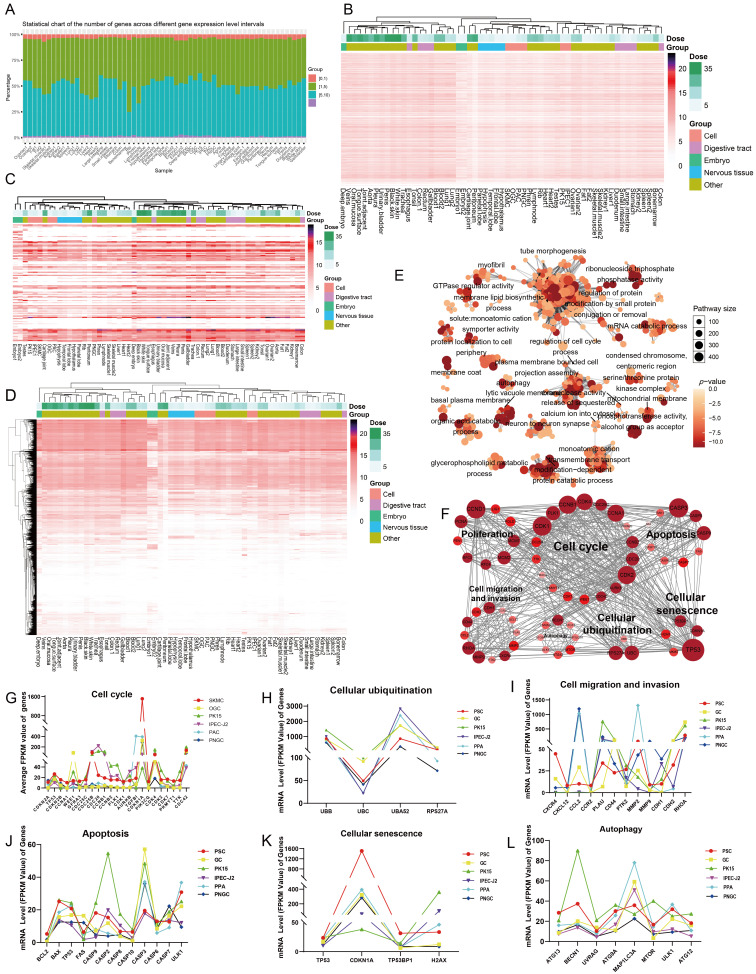
Pig genes exhibit a high degree of expression commonality across tissues and cells. (**A**) Distribution of gene expression levels across 6 porcine cell types and nineteen tissues. (**B**) RNA Expression Profile of Sample Horizontal Clustering Including All Randomly Ordered Genes (35,682). (**C**) Expression Profiles of 112 Randomly Selected, Consecutive Genes from the Expression Profiles of All Genes in (**B**). (**D**) Clustering heatmap showing the expression profiles of 35,682 porcine genes across all samples. (**E**) Functional network of significantly enriched GO terms for ubiquitously expressed genes. (**F**) Interaction network of marker genes associated with common cellular phenotypes, including proliferation, apoptosis, cell cycle, and plasticity. (**G**–**L**) Line charts showing the FPKM values of marker genes related to the cell cycle (**G**), cellular ubiquitination (**H**), migration and invasion (**I**), apoptosis (**J**), cellular senescence (**K**), and autophagy (**L**) in the 6 porcine cell types.

Differences in the RNA expression levels of genes are one of the main causes of variations in the morphology and functions of various cell types. Then, is there a commonality in the RNA expression of genes across different tissues and cells? In response to this, we carried out an analysis of the gene expression characteristics based on the RNA-seq data of the above 43 types of cells and tissues. Except that the number of RNA expression levels of rib within the interval of [1, 5) is a bit large, the distribution quantities of RNA expression levels in other cells and tissues within different intervals are almost the same (Figure 4A). To more intuitively display the RNA expression level of genes in cells, cell lines, and tissues, we performed visualization of the RNA expression levels of all genes (35,682) in the porcine genome. When viewed horizontally from the expression profile, it can be intuitively observed among the 35,682 randomly ordered genes that all genes exhibit high consistency in expression across all samples, with only variations in expression levels. This indicates that genes, whether they express RNA or not, display high similarity across different cells and tissues (Figure 4B). To further demonstrate the universality of gene expression across different cells and tissues, the expression profiles of 112 consecutive genes randomly selected from the whole genome reveal a high degree of similarity in RNA expression of genes across tissues and cells (Figure 4C). The results of horizontal clustering of all samples showed that the three samples of Deep embryo, embryo1, and embryo2 formed an independent branch at the first level of clustering, indicating that the three embryonic samples have the strongest RNA tissue-specificity. In contrast, all other cell and mature tissue samples were clustered into two groups parallel to the embryonic cluster. Notably, cell samples, neural tissues, digestive system tissues, and other tissues exhibited a random interspersed clustering pattern, which suggests that RNA expression has extensive conservation across different cells and tissues. In the longitudinal clustering of all genes, it can be observed that approximately half of the genes express RNA; among these, the gene expression patterns of embryo and Deep embryo remained relatively distinct, while the overall RNA expression abundance of all genes in Cartilage-joint and Peritoneum was the lowest. Despite differences in sequencing depth among all samples, the ranges of their RNA expression profiles were extremely close (Figure 4D). Based on the common characteristics of genome-wide RNA expression, 4117 genes expressed RNA in all 43 types of tissues (Table S2). These basal ubiquitously expressed genes encompass 642 GO terms (Figure 4E and Table S3). The functional signaling pathways enriched in these ubiquitously expressed genes almost cover the signaling pathways of all genes in pigs. There is a strong interaction among the marker genes related to apoptosis, cell cycle, migration, invasion, ubiquitination, and plasticity of the above mentioned six types of cells, with each influencing the other (Figure 4F). Meanwhile, the mRNA expression levels of the marker genes regulating cell ubiquitination, cell senescence, apoptosis, and autophagy present a regular proportion (Figure 4H,I,K). The mRNA expression levels of the genes that regulate cell invasion, migration, and the cell cycle do not exhibit obvious expression patterns and proportions among various cells (Figure 4G,I). However, the mRNA expression level of the gene Cyclin Dependent Kinase Inhibitor 1A (*CDKN1A*) is extremely significantly high in SKMC (Figure 4J). In summary, the RNA expression levels of all genes in the same species exhibit extremely high consistency across different types of cells and tissues.

## 4. Discussion

Since tool cells still retain some similar environmental characteristics of the corresponding tissue sites, they play an important role in studying the functions of the relevant tissues and organs. Among the functional enrichment results of genes related to these 6 types of commonly used porcine tool cells, we obtained a result that is relatively consistent with expectations. Genes that are positively correlated with SKMC are enriched in signaling pathways associated with skeletal muscle and muscle differentiation and development, which exactly reflects the primary functional performance of highly expressed and specifically expressed genes in these cells. Nevertheless, KEGG pathway enrichment analysis reveals that these genes are predominantly enriched in pathways linked to neurological diseases, and individuals with such diseases frequently present with motor dysfunction, including poor coordination, unsteady gait and balance disorders [17]. Based on these findings, we hypothesize that genes involved in neurological disease related metabolic pathways may also influence muscle structure development, thereby contributing to motor abnormalities observed in patients, such as epileptic seizures, spastic hemiplegic gait, scissor gait, and festinating gait [18,19]. For instance, depression, a prevalent neurological disorder, has been reported to be associated with multiple genes, including phosphatase and actin regulator 4 (Phactr4) and NOD-Like Receptor family, Pyrin domain containing 3 (*NLRP3*) [20,21]. Among them, excessive activation of the NLRP3 inflammasome mediates skeletal muscle atrophy [22]. In contrast, the GO and KEGG results for genes significantly correlated with PK15 mainly involve functional signaling pathways related to DNA, RNA, proteins, cell nuclei, and the cell cycle. The substantial enrichment of genes involved in cell cycle regulation may partly explain why PK15 demonstrates the most stable cellular activity among various cell types. Owing to its exceptional tolerance and stability, PK15 is widely utilized in the production of Porcine circovirus type 2 (PCV2) vaccines [23]. It also serves as an important host cell for the preparation of Classical Swine Fever Virus (CSFV) vaccines. Interestingly, despite its use in CSFV research, PK15 is not uniformly or effectively susceptible to CSFV infection, further highlighting its robust stability and defensive capability [24]. As a crucial host in virological studies, PK15 is commonly used for the isolation and in vitro propagation of viruses such as Classical Swine Fever Virus, Pseudorabies Virus, and Porcine Parvovirus, as well as for the production of various veterinary vaccines [25,26]. This widespread use underscores the stability and versatility of PK15 cells. This stability may be attributed to the fact that PK15 is a clonal line derived from PK-2a cells, and its genome exhibits regions of variable aneuploidy and clonal variation [27]. As the largest organ responsible for digestion and absorption in pigs, the small intestine plays a key role in nutrient uptake. The genes significantly associated with IPEC-J2 were primarily enriched in three pathways related to Nicotinamide adenine dinucleotide (NADH) metabolism. NADH, or reduced coenzyme I, participates in cellular material and energy metabolism. It is produced during glycolysis and the citric acid cycle, acting as both a biological hydrogen carrier and an electron donor. Through oxidative phosphorylation on the inner mitochondrial membrane, NADH provides energy for ATP synthesis, and is therefore also referred to as mitochondria [28]. Genes associated with PAC are mainly enriched in signaling pathways related to the development of tubular structures. Based on this result, we speculate that these genes may be related to lipid droplet membrane formation in adipocytes, or they have not been identified in adipocyte related studies, while those associated with PNGC were primarily enriched in pathways linked to immune responses, responses to external stimuli, and the metabolism of certain diseases. The innate immune system within the nervous system is highly diverse, and glial cells serve as key immunoprotective barriers of the central nervous system [29]. Among them, microglia and macrophages are essential immune cells; notably, microglia help mitigate Alzheimer’s disease (AD) and Parkinson’s disease (PD) through inflammatory regulation and phagocytosis of alpha-synuclein (α-Syn) [30].

Based on the above results of gene functional enrichment analysis positively correlated with various cell types, it can be found that the relatively highly expressed and specifically expressed genes of each cell type appear to be the key factors for cells to perform their main functions, and such genes also play crucial roles in many tissues; As genes that serve as relatively highly expressed, tissue-specific genes across various tissues, we propose that such genes should be specifically expressed in one particular tissue or organ and absent in all other tissues. In contrast, genes with relatively high tissue expression show a distinctly dominant expression level in one tissue compared with all other tissues and organs, with a clear, large expression gap. Similar highly expressed and specifically expressed genes exist in numerous other tissues, which are exactly the key genes that maintain the primary function of the corresponding tissue. For instance, the primary function of relatively highly expressed and specifically expressed genes in SKMC is to regulate the differentiation and development of skeletal muscle. Therefore, we suggest increasing research on the highly expressed and specifically expressed genes in each type of cell, which may help identify the genes regulating the primary function of various cells more quickly.

All cells of each animal individual share the same set of genetic material. For ubiquitously expressed genes (i.e., those expressed in all cell types), they are often subjected to interference, knockout, or overexpression to detect their effects on the normal phenotypes of cells, which consequently leads to the repeated research on these genes. Although this study involves pig populations of different breeds, genders and ages, and there are differences in feeding conditions, RNA sequencing depth and time points, the whole genome RNA expression profiles of their digestive tract tissues, visceral organs, nerve tissues, adipose tissues, muscle tissues, reproductive tissues and various tool cells still show high similarity. The main difference among these ubiquitously expressed genes lies in their differences in expression levels among various tissues or cells. These expression differences appear to be shaped by the unique microenvironments of each cell type. Approximately 16,000 genes exhibit low or barely detectable RNA expression levels in all tissue and cell samples, while the RNA expression profile of embryos is significantly different from all other samples. This difference likely reflects the totipotent state of the embryo, which requires extensive transcriptional activation to prepare for subsequent cell differentiation and tissue and organ development. In contrast, mature cells and tissues have highly similar RNA expression ranges, attributed to a large number of ubiquitously expressed genes that mainly undertake cellular basic metabolism and genetic information transmission. Such ubiquitously expressed genes are numerous in mature cells and tissues. For example, let us randomly list some functions of the gene *CDK5RAP3* (Cyclin-Dependent Kinase 5 Regulatory Subunit-Associated Protein 3). According to existing reports, this gene is crucial for maintaining the development of the liver and intestinal Paneth cells [31,32]. The downregulated expression of this gene is associated with renal cell carcinoma [33], and defective *CDK5RAP3* in neurons leads to cerebral hypoplasia [34]. Additionally, *CDK5RAP3* and Ribosomal Protein L26 (RPL26) jointly maintain the stability of cell growth [35]. The ubiquitously expressed genes obtained in this analysis contain a large number of housekeeping genes. The summary and classification of genes expressed in all tissues and cells with stable expression levels just like many housekeeping genes such as *CDK5RAP3* and *GAPDH* will improve gene research efficiency. For instance, numerous experiments focus on evaluating phenotypic marker genes those related to cell proliferation, apoptosis, cell cycle regulation, or cellular plasticity after overexpression or interference of target genes, either in vitro or in vivo. When conducting relevant studies, if the selected target gene is a ubiquitously expressed gene and has been confirmed to regulate cell phenotypes such as proliferation, apoptosis and autophagy in certain cell types of this species, can we consider reducing such repetitive research work in other cell types of the same species? For example, in porcine PK15 and PNGC cell lines, the effects of miR-9828-3p overexpression or knockdown on *JARID2* gene expression levels, as well as the proliferation and apoptosis of these two cell lines, were consistent. There are many such ubiquitously expressed genes that exert the same function in multiple cell types [16]. Likewise, *Hox* genes are known to regulate muscle development across both vertebrates and invertebrates [7]. Some genes even retain identical functions among species within the same subphylum. We speculate that in pigs and other animals, ubiquitously expressed genes are most likely responsible for maintaining the most basic cellular life activities, while tissue-specifically expressed genes and relatively highly expressed genes are most likely responsible for the core functions of the corresponding tissues and organs. Given the large number of variables across all RNA-seq datasets analyzed in this study, the observation of such a high degree of gene expression commonality is remarkable. In this study, it is truly remarkable that the RNA expression levels of all genes in pigs exhibit such high consistency in randomly downloaded multi-tissue datasets. This consistency provides a certain basis for reducing the redundant validation of genes in different cells within the same species and across cells of different species.

## 5. Conclusions

This analysis suggests that highly and specifically expressed genes in each cell type are potentially the key genes responsible for the cell’s primary functions. Further investigation into the uncharacterized genes among them may advance research on gene function. Regarding the RNA expression of genes, the tissue specificity of such expression in embryonic tissues is the highest relative to that in mature tissues and cells, while RNA expression in mature tissues and cells exhibits high conservation and universality.

## Data Availability

The raw RNA sequencing data of this study were derived from the National Center for Biotechnology Information Sequence Read Archive (https://submit.ncbi.nlm.nih.gov/subs/sra/, accessed on 22 April 2025; see Section 2 for specific accession numbers) and the CNGB Nucleotide Sequence Archive (CNSA) of the public data repository of the China National GeneBank Database (CNGBdb) (https://db.cngb.org/cnsa/, accessed on 25 April 2025; accession number: CNP000136). The RNA sequencing data of porcine glial cells and brain tissues were provided by our laboratory. The European Nucleotide Archive (ENA) under accession code PRJNA883198 for the Jin_2023 dataset; the ENA under accession code PRJNA594300 for the Li_2019 dataset; the ENA under accession code PRJNA773967 for the Liu_2022 dataset; the ENA under accession code PRJNA1180182 for the Wang_2024 dataset; RNA-seq data of ovarian granulosa cells and porcine neuroglial cells were provided by our laboratory; the ENA under accession code PRJEB23654 for the Hamill_2018 dataset; the ENA under accession code PRJNA736938 for the Duan_2022 dataset; the ENA under accession code PRJNA1104148 for the Kim_2024 dataset; the ENA under accession code PRJNA1215451 for the Konkuk University_2024 dataset; the ENA under accession code PRJNA562823 for the Sun_2019 dataset; the ENA under accession code PRJNA1224715 for the PLBS_2025 dataset; the ENA under accession code PRJNA854394 for the Li_2022 dataset; the ENA under accession code PRJNA882399 for the Liu_2022 dataset; the China National GeneBank Database (CNGBdb) under accession code CNP0001361 for the Uhlen_2022 dataset; the ENA under accession code PRJNA903765 for the Chen_2023 dataset; the ENA under accession code PRJNA953778 for the Reid_2023 dataset; the ENA under accession code PRJNA271310 for the Hume_2016 dataset.

## Supplementary Materials

The following supporting information can be downloaded at: https://www.mdpi.com/article/10.3390/biom16030448/s1, Table S1: Gene Significance_and_Module Membership. Table S2: Name of ubiquitously expressed genes. Table S3: Ubiquitously expressed genes GO Enrichment Results. Figure S1. Total read counts of RNAs from six cell types. A *p*-value < 0.05 indicates a significant difference with statistical significance.

## Abbreviations

The following abbreviations are used in this manuscript:

SKMC	Skeletal muscle cells
OGC	Ovarian granulosa cells
IPEC-J2	Intestinal porcine enterocyte cell line
PNGC	Porcine neuroglial cells
