# Supplementary material for: Analysis of RNA Expression Specificity and Commonality in Commonly Used Tool Cells and Multiple Tissues of Pigs
Source: Biomolecules. 2026 Mar 17;16(3):448. doi: 10.3390/biom16030448 (PMC13024099; doi:10.3390/biom16030448)
Supplement: Supplementary file 1 [file biomolecules-16-00448-s001.zip › Figure S1.pdf]

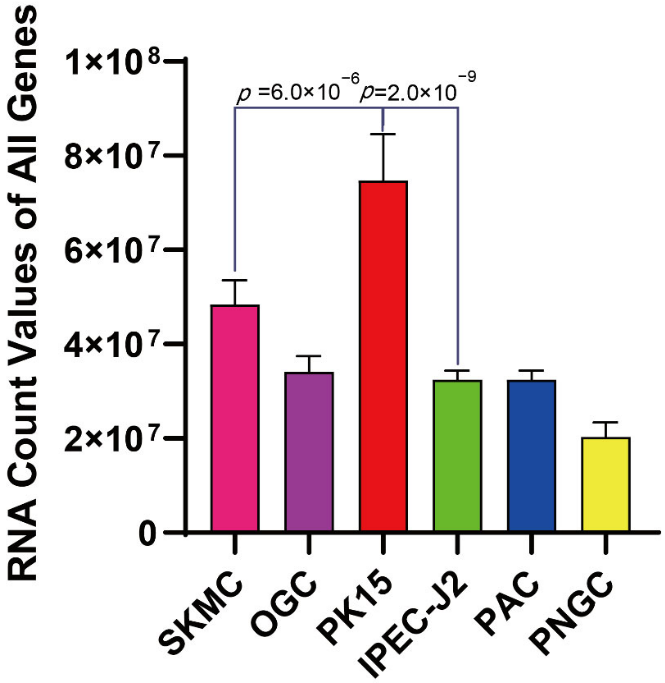

Figure S1. Total read counts of RNAs from six cell types. A *p*-value < 0.05 indicates a significant difference with statistical significance.
